# Physiological Aging Influence on Brain Hemodynamic Activity during Task-Switching: A fNIRS Study

**DOI:** 10.3389/fnagi.2017.00433

**Published:** 2018-01-08

**Authors:** Roberta Vasta, Simone Cutini, Antonio Cerasa, Vera Gramigna, Giuseppe Olivadese, Gennarina Arabia, Aldo Quattrone

**Affiliations:** ^1^Neuroscience Research Center, University Magna Graecia, Catanzaro, Italy; ^2^Department of Developmental Psychology, University of Padova, Padova, Italy; ^3^Institute of Bioimaging and Molecular Physiology, Neuroimaging Research Center, Consiglio Nazionale Delle Ricerche (CNR), Catanzaro, Italy; ^4^Institute S. Anna, Research in Advanced Neurorehabilitation, Crotone, Italy; ^5^Institute of Neurology, Department of Medical and Surgical Sciences, University Magna Graecia, Catanzaro, Italy

**Keywords:** task-switching, physiological aging, functional near-infrared spectroscopy, cognitive control, regression analysis

## Abstract

Task-switching (TS) paradigm is a well-known validated tool useful for exploring the neural substrates of cognitive control, in particular the activity of the lateral and medial prefrontal cortex. This work is aimed at investigating how physiological aging influences hemodynamic response during the execution of a color-shape TS paradigm. A multi-channel near infrared spectroscopy (fNIRS) was used to measure hemodynamic activity in 27 young (30.00 ± 7.90 years) and 11 elderly participants (57.18 ± 9.29 years) healthy volunteers (55% male, age range: (19–69) years) during the execution of a TS paradigm. Two holders were placed symmetrically over the left/right hemispheres to record cortical activity [oxy-(HbO) and deoxy-hemoglobin (HbR) concentration] of the dorso-lateral prefrontal cortex (DLPFC), the dorsal premotor cortex (PMC), and the dorso-medial part of the superior frontal gyrus (sFG). TS paradigm requires participants to repeat the same task over a variable number of trials, and then to switch to a different task during the trial sequence. A two-sample *t*-test was carried out to detect differences in cortical responses between groups. Multiple linear regression analysis was used to evaluate the impact of age on the prefrontal neural activity. Elderly participants were significantly slower than young participants in both color- (*p* < 0.01, *t* = −3.67) and shape-single tasks (*p* = 0.026, *t* = −2.54) as well as switching (*p* = 0.026, *t* = −2.41) and repetition trials (*p* = 0.012, *t* = −2.80). Differences in cortical activation between groups were revealed for HbO mean concentration of switching task in the PMC (*p* = 0.048, *t* = 2.94). In the whole group, significant increases of behavioral performance were detected in switching trials, which positively correlated with aging. Multivariate regression analysis revealed that the HbO mean concentration of switching task in the PMC (*p* = 0.01, β = −0.321) and of shape single-task in the sFG (*p* = 0.003, β = 0.342) were the best predictors of age effects. Our findings demonstrated that TS might be a reliable instrument to gather a measure of cognitive resources in older people. Moreover, the fNIRS-related brain activity extracted from frontoparietal cortex might become a useful indicator of aging effects.

## Introduction

Task-switching paradigm is a well-known and validated tool for exploring executive control processes and neural correlates of cognitive cost and it is often used to assess age-related executive deficits (Wilckens et al., 2017). Typically, this paradigm requires the repetition of the same task over a variable number of trials (i.e., repetition trials) and the rapid alternation between two different tasks at some point of the trials sequence (i.e., switch trials). For each trial a reaction time (RT) is registered. Switch cost refers to the finding that performance is slower (longer RTs) and less accurate on switch trials than repeated trials and is thought to reflect the executive processes required to deactivate the task set relevant on the previous trial and to activate the currently relevant task set (Monsell, 2003). Task-switching performance may be improved using task cues, which provide valid information about the upcoming target and allow for time to prepare for a given trial (Schapkin et al., 2014). Task cues “effect” is associated to maintaining and reconfiguration processes of a task set in working memory (Wilckens et al., 2017).

In the last decades several studies have highlighted the fundamental role of the dorsolateral and ventrolateral prefrontal cortex (dlPFC, vlPFC), the supplementary and pre-supplementary motor areas (SMA, pre-SMA) and the superior and inferior lobules of the parietal cortex in task-switching (Dove et al., 2000; Braver et al., 2003; Brass and von Cramon, 2004; Wager et al., 2004; Ruge et al., 2005; Badre and Wagner, 2006; Crone et al., 2006; Slagter et al., 2006; for reviews see Ruge et al., 2013; Jamadar et al., 2015).

Physiological aging modulation of these brain areas in switching task has been extensively investigated by using both structural and functional advanced magnetic resonance imaging (DiGirolamo et al., 2001; Milham et al., 2002; Gold et al., 2010; Zhu et al., 2014; Hakun et al., 2015; Eich et al., 2016; Jolly et al., 2017), suggesting that age-related changes in behavioral performance are associated with changes in neural patterns of activation. Specifically, older adults show a less specific cerebral activation and the recruitment of additional frontal regions that are not activated in younger adults (DiGirolamo et al., 2001; Milham et al., 2002; Gold et al., 2010).

Although these neuroimaging modalities are highly effective and reliable, these are also very expensive and invasive. fNIRS is a non-invasive neuroimaging technique that enables to investigate brain hemodynamic) with reasonable temporal and spatial resolution, quantifying task-related changes in oxygenated hemoglobin (HbO), and deoxygenated hemoglobin (HbR) concentrations (Scholkmann et al., 2014); crucially for our purposes, fNIRS provides a remarkable added value in cognitive neuroscience because it allows to gather information on cortical activity in those overcoming some limitations imposed by other neuroimaging techniques, thereby increasing the ecological validity of the tasks used to test participants (Cutini et al., 2012, 2014; Cutini and Brigadoi, 2014). The advantages of being non-invasive, portable and relatively low susceptible to motion artifacts than other neuroimaging techniques, give NIRS a strong ecological validity for use in situated cognition paradigms (Ferreri et al., 2014).

For this reason, this neuroimaging technique has gained growing interest in the last 10 years, particularly in the field of cognitive aging (Agbangla et al., 2017), focusing on several domains cognitive function such as language (Scherer et al., 2012; Amiri et al., 2014), episodic memory (Ferreri et al., 2014), executive functions (verbal fluency; Herrmann et al., 2006; Kahlaoui et al., 2012; Obayashi and Hara, 2013), working memory (Vermeij et al., 2012, 2014a,b, 2016), inhibition and cognitive flexibility (Schroeter et al., 2003; Laguë-Beauvais et al., 2013; Hagen et al., 2014; Müller et al., 2014).

In the context of attentional control functions, very few studies have monitored brain hemodynamic changes during the task-switch execution by using fNIRS technology (Cutini et al., 2008; Laguë-Beauvais et al., 2013). Among them, just one study has evaluated physiological aging effect on brain areas modulation during inhibition and switching tasks (Laguë-Beauvais et al., 2013). The authors compared fNIRS-related functional brain activation patterns in the prefrontal cortex in older and younger adults during a modified Stroop task with interference and switching conditions, by using a classical univariate statistical approach. Conversely, the lesson learnt from one decade of neuroimaging studies provides consistent evidence of the advantage of multivariate analysis of moving from group-level statistical results to a full description of a biologic phenomenon (Habeck, 2010).

For this reason, our aim was to develop an ecologically sound and easily applicable mean to assess both behavioral and neurofunctional age-related changes in switching task by using functional near-infrared spectroscopy (fNIRS) and a multivariate statistical approach. In this study, we investigated the hemodynamic response in the frontoparietal areas during the execution of a task-switching paradigm by means of fNIRS on a population of healthy participants, and we characterized the selective influence of physiological aging on brain hemodynamic response by using multiple linear regression.

Within the present framework, we sought to explore whether the recruitment of additional frontal regions is a pervasive phenomenon that can be observed in the vast majority of the frontal lobe or if it is restricted to a subset of regions. In this regard, multiple linear regression gave us the chance to observe a possible dissociation between those regions that might help to compensate the age-related cognitive decline and those regions that might be indeed less activated in elderly participants.

## Materials and methods

Participants were recruited from University of Catanzaro, Polyclinic “Magna Graecia,” community recreational centers and hospital personnel through local advertisements. Inclusion criteria were: (1) no evidence of dementia or depression symptoms according to DSM-V criteria; (2) no use of antidepressant, anxiolytic, or antipsychotic drugs that could affect cerebral blood flow; (3) right- handedness; and (4) absence of chronic medical conditions (heart disease, hypertension, or diabetes); According to these criteria, 38 right-handed healthy volunteers (21 males and 17 females, in the age range of 19–69 years, mean age = 37.87 ± 14.94 years) were considered eligible for this study. All participants had normal or corrected to normal vision, and normal color vision. All the participants gave written informed consent. The study was approved by the Ethical Committee of the University “Magna Graecia” of Catanzaro, according to the Helsinki Declaration.

### Experimental procedure

The experiment was carried out in a sound-attenuated and dimly lit room. Participants were seated in a comfortable chair while performed a color-shape task-switching paradigm (Hakun et al., 2015), that was designed using E-prime 3.0 software (Schneider et al., 2012) (Psychology Software Tools, Pittsburgh, PA). The synchronization between fNIRS recording and timing of stimulation was performed through the RS232 serial port communication. The stimuli consisted of two possible shapes (circle or square), in one of two possible colors (red or blue), presented on a computer screen. Participants were asked to hold the index and middle fingers of the right hand on the “left” and “right” arrows keys of the computer keyboard throughout the entire experiment, respectively.

At the beginning of each trial, an instructional cue was given to participant (the word “color” or “shape”), which was displayed for 150 ms. Upon the presentation of a color stimulus, participants had to press “right” key in response to “red,” or the “left” key in response to “blue.” Upon the presentation of a shape stimulus, participants had to press “right” key for “square,” or the “left” key for “circle.” Each stimulus was presented for a maximum of 3,000 ms, and replaced with a black screen upon detection of a response (with a duration randomly varying from 8 to 10 s). Then a 200 ms central fixation (plus-sign) signaled the start of the next trial. Before detection, participants received task instructions and practiced for each condition.

**Figure 1:**
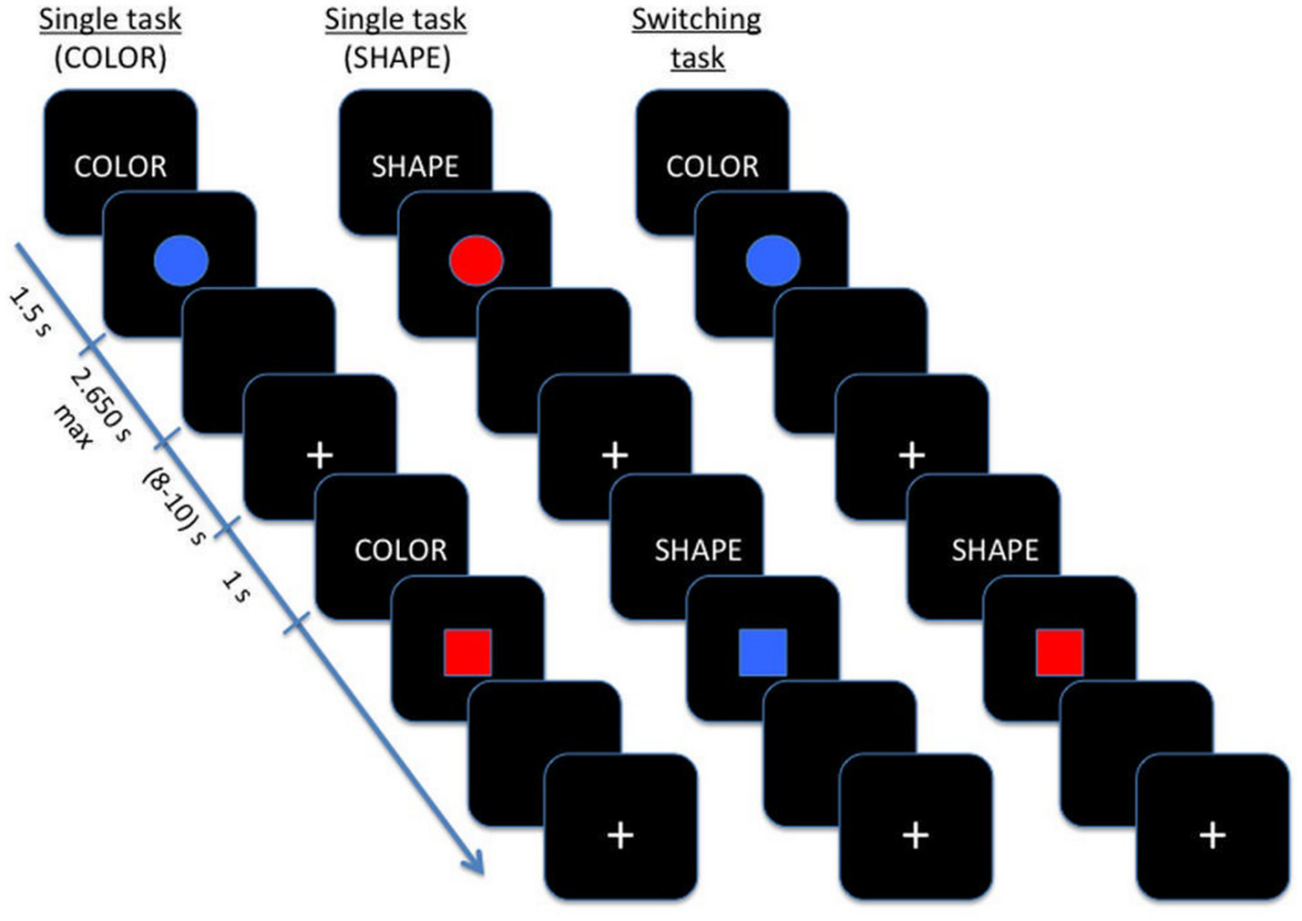
Schematic illustration of Color-shape task-switching paradigm.

As showed in Figure 1, the task was composed by three main blocks: (a) in the color block, participants were required to distinguish between red and blue stimuli; (b) in the shape block, participants were asked to judge when the stimulus was a circle or a square; (c) in the switching block, shape and color stimuli were shown alternatively to patients. Blocks (a) and (b) were regarded as single blocks, whereas switching blocks included repetition and switch trials.

In single blocks, participants performed a sequence of 20 experimental trials with the same instructional cue (color or shape) repeated on each trial, while during switching blocks the color and shape tasks were presented pseudo-randomly, with an equal number of repeating/ switching in consecutive trials (40 switch trials and 40 repetition trials).

RTs and the percentage of correct response were calculated for single blocks, repetition and switch trials.

An independent two-sample *t*-test was performed in order to evaluate behavioral differences between groups in task-switching performances. We calculated the Cohen's d (Cohen, 1998) as a measure of the effect size, which indicates the magnitude of mean differences (using the estimated marginal means) in *SD* units.

On the whole group, an ANOVA analysis was carried out on median RT values and the percentage of correct responses in order to highlight possible differences among single blocks, repetition and switch trials. Moreover, a simple regression analysis was performed in order to evaluate the effect of aging on task-switch performances.

### fNIRS probe location and data acquisition

A 52-channel NIRS machine (ETG-4000 Optical Topography System; Hitachi Medical Co., Japan) working with two different wavelengths (695 and 830 nm) and a sample frequency of 10 Hz was used to measure relative changes of absorbed near-infrared light during color-shape task switching.

**Figure 2:**
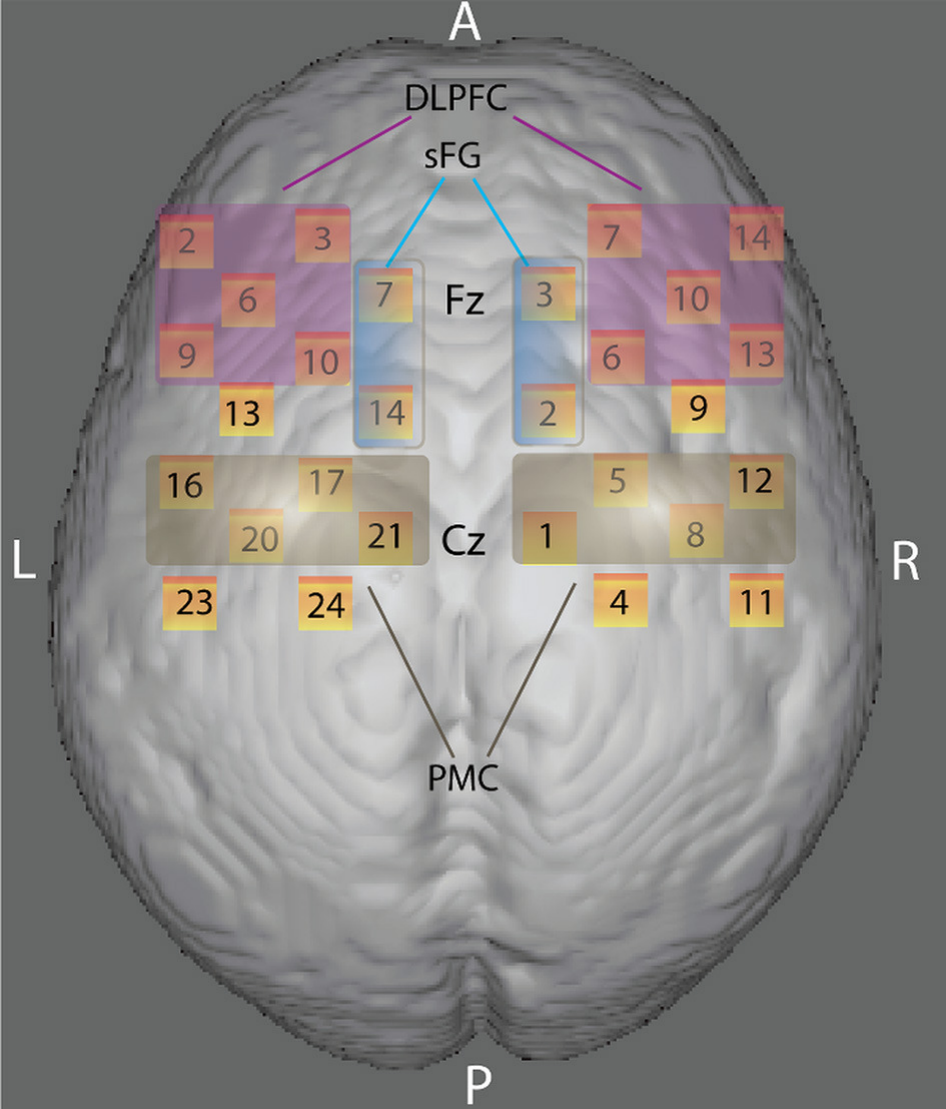
Anatomical ROIs localization. Rendering of the skull surface showing the detection channels to record brain activity in the right and left dorso-lateral prefrontal cortex (DLPFC), the right and left dorsal premotor cortex (PMC), and the right and left dorso-medial part of the superior frontal gyrus (sFG) during color-shape task-switching paradigm, according to 10/20 system.

Two “4 × 4” measurement grids were attached to a regular swimming cap. Eight emitters and eight detectors -for a total of 24 measurement channels for each hemisphere- were used (Figure 2) and each source/detector pair at a distance of 3 cm. According to the international 10/20 system, channel grids were placed to cover following ROIs for each hemisphere: the dorso-lateral prefrontal cortex (DLPFC), the dorsal premotor cortex (PMC) and the dorso-medial part of the superior frontal gyrus (sFG) (see “Data Sheet 1” for an example of photon migration and penetration depth). Figure 2 shows that dorso-medial part of sFG, DLPFC, and PMC were bilaterally covered by the present probe spatial arrangement.

### fNIRS data analysis

A preliminary visual inspection of the fNIRS intensity signal time-course of each source-detector pair was used to detect the presence of physiological activity and to test fNIRS signal quality.

Noisy source-detector pairs were manually discarded on the base of absence of physiological activity in both 830 and 695 nm signals. Channels that visually showed movement artifacts were excluded from the analysis. A moving average method with a window width of 5 s was used to identify and remove any short-term movement artifacts. Raw fNIRS data were converted into optical density changes and then bandpass filtered between 0.005 and 0.5 Hz, to remove low frequency drifts signal components and cardiac fluctuations interferences. The relative changes in the concentration of HbO, HbR, and total hemoglobin (HbT) were estimated according to changes in the optical properties of the light using the Beers-Lambert law (Cope and Delpy, 1988; Delpy et al., 1988).

Each trial was baseline corrected by subtracting the mean intensity of the optical signal recorded during the 2 s preceding trial onset from the overall hemodynamic activity.

Then HbO and HbR mean concentrations during vascular response were calculated for each subject and task in all channels of interest from standardized grand average waveform (z-score).

### Statistical analysis

Two different statistical approaches were used to evaluate the age-related influence on task-switching activity. Initially, we considered aging effect as discrete factor. For this reason, we grouped healthy sample in young and elderly populations. Age ≥50 years was used as cut-off for defining elderly people, since several studies demonstrated the presence of physiological neurodegenerative processes starting after the so-called: non-elderly adult phase (18–50 years; Pieperhoff et al., 2008; Terribilli et al., 2011). Twenty-seven young participants (mean age = 30.00 ± 7.90 years) and 11 elderly participants (mean age = 57.18 ± 9.29 years) were matched for gender (Chi-square test, *p* < 0.05) and education level (*t*-test, *p* < 0.05).

Next, simple regression or multivariate regression analyses considering the impact of aging effect as a continuous factor were employed. Statistical analyses were performed with SPSS Version 12.0 (https://www.ibm.com/software/products/it/spss-statistics). Assumptions for normality were tested for all continuous variables by using the Kolmogorov–Smirnov test. Unpaired *t*-test and analysis of variance were employed appropriately for behavioral data. Finally, Pearson correlation analysis was used for evaluating the relationship between age and task performance. For all statistical analyses, a *p*-level of 0.05 was considered to be significant. Moreover, Cohen's *d* as a measure of the effect size was also calculated (Cohen, 1998).

A similar approach was used for fNIRS data. We started with an independent two-sample *t-*test to evaluate differences between groups in hemodynamic activation (HbO and HbR mean concentration) within ROIs. Next, to evaluate how physiological aging could selectively influence brain hemodynamic response, we performed a multiple linear regression, according to the model: age = β x (predictors) + constants. In particular, with the aim of quantifying the relative contribution to aging of each task (color single-task, shape single-task, repetition trials, switch trials), each hemodynamic parameter (HbO and HbR mean concentrations) and each ROI (for both the hemispheres separately), we performed a regression analysis using a multiple linear model including all predictors.

## Results

### Demographic data

Demographic features of all subjects are summarized in Table 1. No differences were detected in gender (*p* = 0.876) and education level (*p* = 0.312) between young and elderly participants.

**Table 1:** Participant's demographic and behavioral data.

	**Young participants**	**Elderly participants**	***p*-value**
**DEMOGRAPHIC DATA**			
Age (years)	30.00 ± 7.90	57.18 ± 9.29	<0.01^§^
Gender (%M)	55.56%	54.55%	0.876^*^
Education Level (years)	12.12(5–18)	10.44(5–13)	0.312^§^
**BEHAVIORAL PERFORMANCES**			
**Reaction time (ms)**			
Color-single task	560.91 ± 149.71	802.57 ± 196.24	<0.01^§^
Shape-single task	581.66 ± 126.06	774.84 ± 239.60	0.026^§^
Repetition trials	748.77 ± 215.05	972.08 ± 226.11	0.012^§^
Switching trials	823.56 ± 249.20	1035.82 ± 244.81	0.026^§^
**PERCENTAGE OF CORRECT RESPONSES**			
Color-single task	96.85 ± 4.63	95.45 ± 6.11	0.447^§^
Shape-single task	94.07 ± 17.27	96.36 ± 4.52	0.669^§^
Repetition trials	92.50 ± 10.74	88.35 ± 12.99	0.316^§^
Switching trials	90.83 ± 11.27	88.86 ± 14.42	0.655^§^

* *χ^2^*.

§ *Unpaired two-sample t-test*.

### Behavioral data

An independent two sample *t*-test revealed significantly longer reaction time (RT) for elderly compared to young participants in both color- (*p* < 0.01, *t* = −3.67; Cohen's d = −1.21, effect size = 0.52) and shape-single tasks (*p* = 0.026, *t* = −2.54; Cohen's d = −0.84, effect size = 0.39) as well as switching (*p* = 0.026, *t* = −2.41; Cohen's d = −0.79, effect size = 0.37) and repetition trials (*p* = 0.012, *t* = −2.80; Cohen's d = −0.92, effect size = 0.42) (see Table 1). No significant differences between groups were detected in the correct response percentage (Table 1).

**Figure 3:**
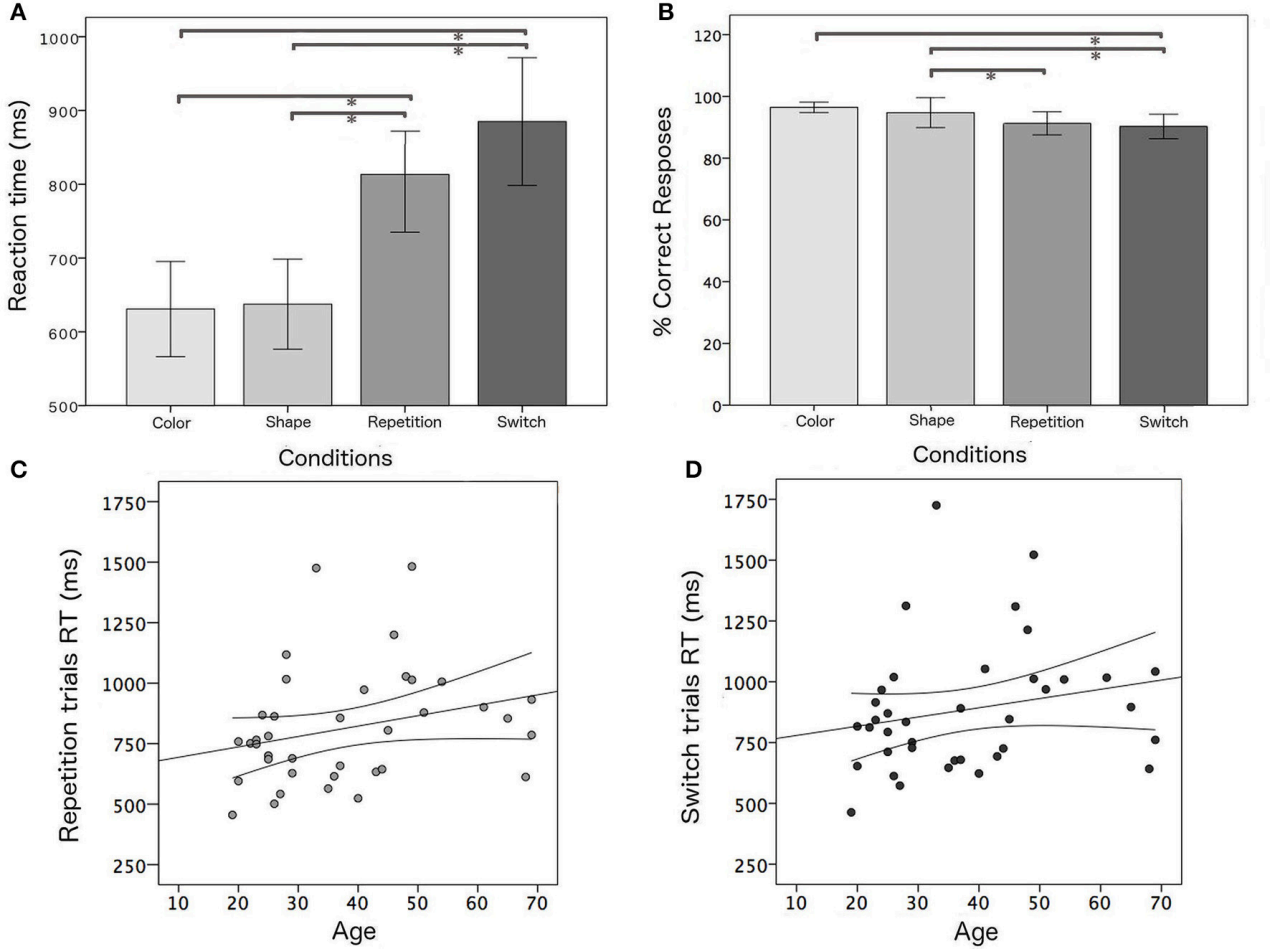
Behavioral results: **(A,B)** mean RTs and mean percentage of correct responses for all task conditions; positive correlation between age and RT and confidence bounds (95%) for **(C)** repetition and **(D)** switching trials, respectively. ^*^Significant results of post-hoc test among task conditions in ANOVA analysis for the whole group (*p* < 0.05).

An ANOVA analysis was carried out on median RT values and the percentage of correct responses of the whole group, considering the four task conditions (color, shape, switch, and repetition trials). As expected, switch trials were associated with longer RTs (*F* = 12.4, *p* < 0.001) and worse accuracy (*F* = 5.6; *p* = 0.001) with respect to single blocks (Figure 3). A simple regression analysis was performed in order to evaluate the effect of aging on switch trials. As expected, performance was positively correlated with age for repetition (*r* = 0.378; *p* = 0.019) and switching trials (*r* = 0.316; *p* = 0.045).

### fNIRS data

An independent two-sample *t*-test revealed differences in cortical activation between young and older participants for HbO mean concentration of switching task in the left PMC (*p* = 0.048, *t* = 2.94; effect size = 0.44 and Cohen's d = 0.97).

**Figure 4:**
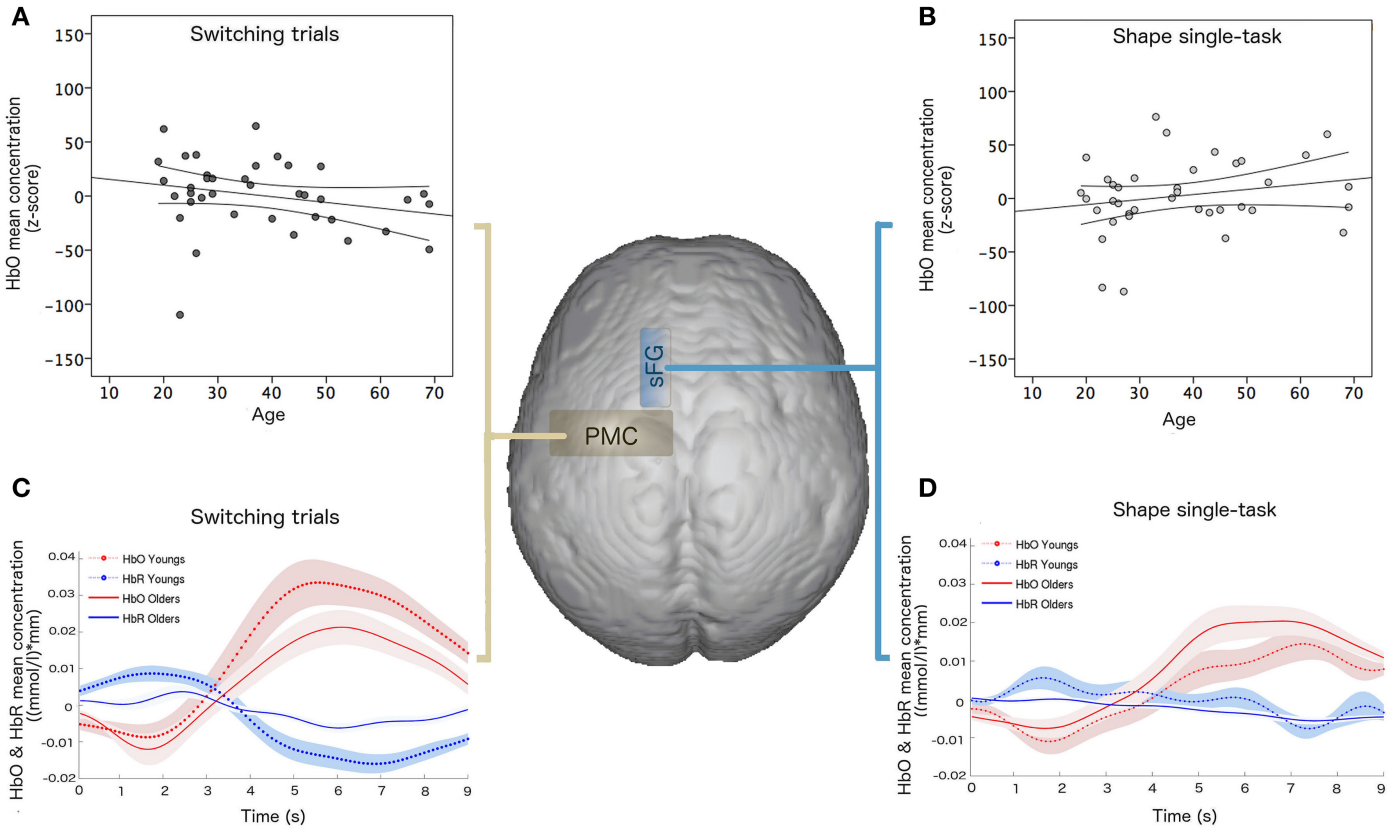
Linear regression results of hemodynamic data and age: HbO mean concentration ((mmol/l)^*^mm) for **(A)** switching task in the PMC and **(B)** shape single-task in the sFG; hemodynamic response function and confidence bounds (95%) of young and old participants for **(C)** switching task in the PMC and **(D)** shape single-task in the sFG.

The multiple linear regression analysis, performed on the whole sample, highlighted that age variance explained by the linear model was about 80% (*R*^2^ = 0.806) and the best age predictors were HbO mean concentration for shape single-task in the sFG (*p* = 0.003, β = 0.342) and HbO mean concentration for switching task in the PMC (*p* = 0.01, β = −0.321) (Figure 4).

## Discussion

In the last decades, neuroimaging studies have been particularly focused in understanding the neurofunctional bases of physiological aging effects on cognitive processes. In particular, functional neuroimaging studies have shown that cognitive control processes involve a broad network centered on frontoparietal areas (e.g., Corbetta and Shulman, 2002; Dosenbach et al., 2008), which are thought to subserve underlying different cognitive operations (D'Esposito et al., 1995; Duncan et al., 1996; Posner and DiGirolamo, 1998). Age-related worsening in behavioral performance is associated with changes in neural patterns of activation, involving the under-recruitment of task-specific regions (deactivations and a decreased spatial extent of activation), hemispheric lateralization, and the recruitment of additional brain areas, especially of frontal regions (DiGirolamo et al., 2001; Milham et al., 2002; Gold et al., 2010). This increased frontal activation has led to opposing interpretations: evidence of an adaptive positive compensatory mechanism in order to preserve cognitive functioning (Reuter-Lorenz and Cappell, 2008; Davis et al., 2009; Reuter-Lorenz and Park, 2014) or age-related brain dysfunction (Colcombe et al., 2005; Rypma et al., 2005, 2006; Zarahn et al., 2007; Stern, 2009; Gold et al., 2013; Zhu et al., 2015).

The most common tasks used to define cognitive reserve in elderly people are Go/NoGo (inhibitory control), n-back (working memory), and task-switching (cognitive control). Age-related alterations in brain activation tend to be especially pronounced on tasks that emphasize cognitive control processes (Drag and Bieliauskas, 2010). As a consequence, among these, task-switching has been extensively used to evaluate physiological aging influence on executive deficits (DiGirolamo et al., 2001; Milham et al., 2002; Gold et al., 2010; Zhu et al., 2014; Hakun et al., 2015; Eich et al., 2016; Jolly et al., 2017). However, the existing knowledge on this research field has been mainly achieved by advanced neuroimaging methods, as Positron Emission Tomography (PET) (Berry et al., 2016) structural MRI (Zhu et al., 2014; Jolly et al., 2017) and functional MRI (DiGirolamo et al., 2001; Milham et al., 2002; Gold et al., 2010; Hakun et al., 2015; Eich et al., 2016).

Albeit these conventional neuroimaging modalities have proven to be effective and reliable, their well-known limitations (very expensive, invasive, and with several constraints for patients with physical limitations) make them unsuitable for a large scale application. fNIRS is a non-invasive neuroimaging technique able to investigate *in vivo* brain hemodynamic (Villringer et al., 1993) with reasonable temporal and spatial resolution, quantifying task-related changes in oxygenated hemoglobin (HbO) and deoxygenated hemoglobin (HbR) concentrations (Cutini et al., 2014).

By the advantages of being non-invasive, more ecological than conventional neuroimaging methodologies and able to investigate *in vivo* brain hemodynamic (Villringer et al., 1993) with reasonable temporal and spatial resolution, fNIRS technology has been recognized as a suitable tool for application in the field of cognitive aging (Agbangla et al., 2017).

The most consistently reported pattern of age-related differences in brain activation is the increased high involvement of the prefrontal cortex. This overactivation in older adults is often interpreted as a compensatory mechanism when it is concomitant with preserved cognitive performance. Recent results of fNIRS studies on working memory (Vermeij et al., 2012, 2014a,b, 2016) are in agreement with this model, showing that higher activation at a high cognitive load was predictive of higher behavioral improvements, whereas relatively higher prefrontal recruitment at a low cognitive load was related to worse behavioral performance and improvement.

In addition, to the best of our knowledge only one study (Laguë-Beauvais et al., 2013) monitored age-related modulation on task switch using fNIRS during a modified Stroop task. Their univariate statistical approach also confirmed that the two executive processes of interference and switching are associated with distinct patterns of prefrontal activation and that both these patterns appear more spread out in the PFC of older adults.

Our work aimed at overcoming the intrinsic limit of univariate statistic by combining the well-known task-switching paradigm and the fNIRS technology by a multivariate statistical approach. Indeed, we were able to disentangle the relative contribution of age-related functional alterations of frontoparietal areas and to evaluate a possible dissociation between different mechanisms (deactivations or hyperactivation) that those regions adopt to compensate the age-related cognitive decline.

In particular, our behavioral data confirms previous evidence of the importance of the task-switching in defining cognitive cost. Moreover, this greater cognitive demand correlates with age, confirming that aging effect can be captured during specific cognitive tasks. In addition, fNIRS confirms that this effect also occurs at the neurobiological level, with an increase in functional activity of the frontal areas. Although the association between sFG activity and aging has been found in other neuroimaging studies (DiGirolamo et al., 2001; Milham et al., 2002; Gold et al., 2010; Zhu et al., 2014, 2015; Hakun et al., 2015; Berry et al., 2016; Eich et al., 2016; Jolly et al., 2017), the new finding which merits to be highlight is the opposite trend in the correlation between PMC activity (HbO and HbR mean concentrations) and aging. Although surprising, this result may be explained by the fact that age-related cognitive decline is associated to an increase in compensatory functional activity and a simultaneous decreased cortical activity of other regions. In particular, it has been demonstrated that during working memory with increasing task load, older adults showed decreased connectivity and ability to suppress activity in other brain regions. The deactivation of other strictly connected brain regions is essential for the correct execution of cognitively demanding tasks (Sambataro et al., 2010). The positive correlation between age and HbO concentration change in the sFG during the single task could be explained in terms of an additional effort required by working memory process in older people. This finding is consistent with the results of a neuropsychological study (du Boisgueheneuc et al., 2006), which found that patients with a left sFG lesion exhibited a working memory deficit when compared with all control groups.

It is worth noting that the present probe arrangement did not include short separation channels, which are typically employed to eliminate systemic, task-dependent physiological oscillations that might create a confounding factor when evaluating the task-evoked brain hemodynamic response (Tachtsidis and Scholkmann, 2016). This issue is caused by a specific reason: beside capturing hemodynamic variations related to cortical activity, the signal from fNIRS channels with a standard source-detector distance (e.g., 3 cm) is contaminated with superficial, physiological hemodynamic fluctuations (e.g., heartbeat and Mayer's waves), located both in the vasculature of the layers overlaying the brain and in the brain itself (Caldwell et al., 2016). Given that the source-detector distance is inversely related to the proportion of photons reaching the cortex, short-separation channels enable to measure the same global, superficial hemodynamic fluctuations visible in standard channels, while also being insensitive to brain activity (Brigadoi and Cooper, 2015); indeed, confounding effects from extracerebral contamination and systemic factors are eliminated by regressing out the signal obtained from short-channels from the one observed in the standard channels. This procedure assures that the activity found in standard channels can be safely attributed to brain activation. Although with the present arrangement, we cannot completely rule out the presence of a physiological contamination in our results, two aspects deserve careful consideration. The first one concerns the design of the experimental protocol: the stringent control condition provided by repetition trials makes unlikely that the different hemodynamic pattern between switch and repetitions trials can be attributed to physiological oscillations; the experimental paradigm was specifically to have just one difference (i.e., the reconfiguration of task-set) between switch and repetition trials. The same line of reasoning has been recently highlighted in theoretical works (e.g., Scholkmann et al., 2013; Tachtsidis and Scholkmann, 2016) and it can be appreciated in recent fNIRS studies on clinical populations (e.g., Cutini et al., 2016). Second, the hallmark of extracerebral contamination is the ubiquitous presence in all the channels, thereby implying that all the regions should show the same hemodynamic pattern; crucially, in our results we observed a clear functional dissociation between PMC and sFG. Taken together, these two observations strongly suggest that the hemodynamic activity found in the present study is mainly driven by cortical activation.

In conclusion, we might speculate that the two active regions found with fNIRS are both bound to physiological aging but they might be representative of two distinct cognitive processes that are partially dissociable.

## Author contributions

RV: analyses and interpretation of the data, statistical analysis and drafting/revising the manuscript, final approval of the version to be published; SC: study concept and design, analyses and interpretation of the data, statistical analysis, and drafting/revising the manuscript, final approval of the version to be published; AC: study concept and design, data collection and interpretation and drafting/revising the manuscript, final approval of the version to be published; VG: data collection, analysis and interpretation, and drafting/revising the manuscript, final approval of the version to be published; GO: data collection and final approval of the version to be published; GA and AQ: drafting/revising the manuscript, final approval of the version to be published.

### Conflict of interest statement

The authors declare that the research was conducted in the absence of any commercial or financial relationships that could be construed as a potential conflict of interest.
