# Supplementary material for: Physiological Aging Influence on Brain Hemodynamic Activity during Task-Switching: A fNIRS Study
Source: Front Aging Neurosci. 2018 Jan 8;9:433. doi: 10.3389/fnagi.2017.00433 (PMC5767724; doi:10.3389/fnagi.2017.00433)
Supplement: Supplementary file 1 [file DataSheet1.doc]

***Probabilistic Model of Photon Migration through the Head and Sensitivity Analysis***

Properly interpreting fNIRS data requires detailed knowledge of the measurement’s spatial sensitivity profile. Question about sensitivity and penetration depth in complex tissues like cerebral must rely on numerical approaches. There are two general categories: (1) approaches based on finite element (FE) and finite difference (FD) analysis or (2) Monte Carlo simulations (**Strangman et al., 2013)**.

Scientific community usually models the photon migration through the head using a Monte Carlo approach in order to evaluate which of the channels of the specific optodes configuration are more sensitive to the target region.

We computed brain sensitivity profile for each source/detector pair adopting a realistic model of light propagation in biological tissues (Monte Carlos simulations) (**Boas et al, 2002**). This method simulates photon transport and migration in head tissues, taking into account the specific anatomy of each patient.

We used Monte Carlo eXtreme, or MCX [http://mcx.sourceforge.net/cgi-bin/index.cgi], a Monte Carlo simulation software for time-resolved photon transport, to define a probabilistic path of photons through the head. Details about the Monte Carlo code can be found in Fang et al. (**Fang et al., 2009**). It requires a realistic multilayered head model (scalp, skull, CSF, gray matter (GM) and white matter (WM)), segmented from the anatomical T1-weighted MR image of the subject using Freesurfer (**Dale et al., 1999; “FreeSurfer,” http://freesurfer.net/**).

Absorption and scattering coefficient values at 830 nm associated with each tissue type constituting the segmented anatomical head model used for simulation, were available from literature (**Machado et al., 2014**) and showed in Table 1. Tissue anisotropy coefficient equal to 0.9 and refractive indices of air and tissue, set to 1.0 and 1.37 respectively, were considered in Monte Carlo simulation.

A slice of sensitivity profile at 830 nm for two specific ROIs (Inferior Frontal Gyrus (A) and Premotor Cortex (B)) where we found significant results were registered on one subject-specific anatomical MRI and depicted in Figure 1.

| Tissues | 830 nm |
| --- | --- |
| Scalp | 0.0191/8.25 |
| Skull | 0.0136/10.75 |
| CF (Cerebrospinal Fluid) | 0.0026/0.125 |
| GM (Gray Matter) | 0.0186/13.875 |
| WM (White Matter) | 0.0186/13.875 |

Table 1 Absorption/scattering coefficients (mm^-1^) at 840 nm used for the Monte Carlo simulations (Machado et al., 2014).


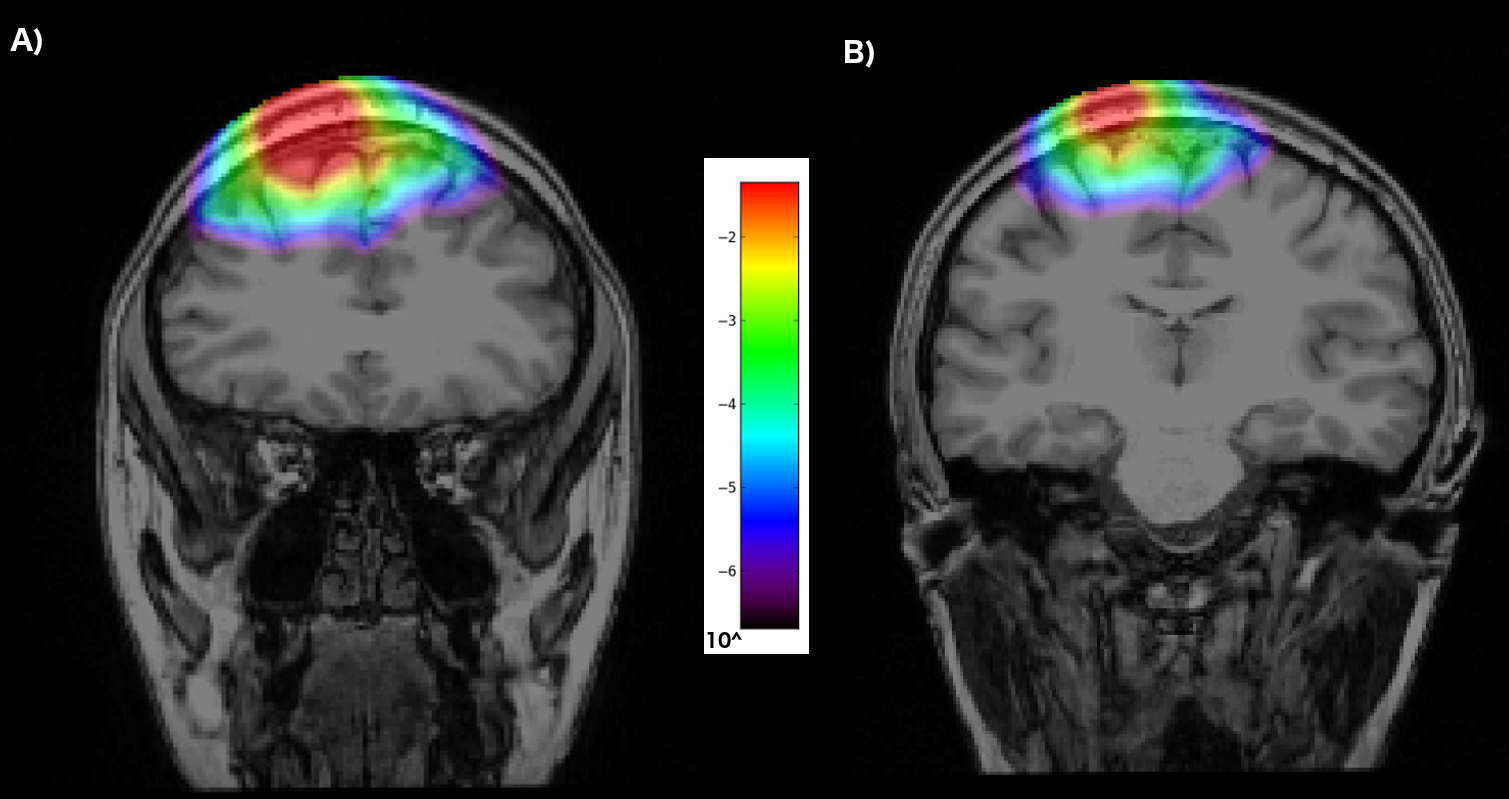


*Figure 1: Coronal view of photon sensitivity profile (color map of sensitivity matrix coefficients) of two source/detector pairs that cover the (A) inferior frontal gyrus and (B) the premotor cortex regions, determined by the propagation of light emanated from a source and recorded by a detector. The color scale depicts exponential values of the sensitivity matrix.*

The results confirmed that our probe spatial configuration was suitable to the target region and the experimental protocol. Indeed, our results are in agreement with relative sensitivity values that are depicted in the paper of Strangman and collegues (**Strangman et al., 2013**).

Further studies need to be performed in order to assess the effect of cerebral cortex folding geometry and on source-detector separation (LSD) on light propagation. Recently, simulation studies based on head models containing a cerebrospinal fluid (CSF) layer have demonstrated that light propagation is highly affected by the presence of CSF. Because the cerebral surface is folded as gyri and sulci filling with CSF, it is probable that the cerebral cortex folding geometry is also important for light propagation in the head. Possibly, the gyri and sulci might change the spatial distribution of sensitivity.

**Additional References**

1. Strangman, G.E., Li, Z., Zhang, Q. (2013) Depth sensitivity and source-detector separations for near infrared spectroscopy based on the Colin27 brain template. PLoS One 8(8), e66319. doi: 10.1371/journal.pone.0066319.
2. Boas, D., Culver, J., Stott, J., Dunn, A. (2002) Three dimensional Monte Carlo code for photon migration through complex heterogeneous media including the adult human head. Opt Express 10,159–170.
3. Fang, Q. and Boas, D.A. (2009) Monte Carlo Simulation of Photon Migration in 3D Turbid Media Accelerated by Graphics Processing Units. Opt Express 17(22), 20178-20190.
4. Dale, A. M., Fischl, B., and Sereno, M. I. (1999) Cortical surface-based analysis. I. Segmentation and surface reconstruction. NeuroImage 9(2), 179–194.
5. “FreeSurfer,” http://freesurfer.net/ (17 April 2015).
6. Machado, A., Marcotte, O., Lina, J.M., Kobayashi, E., Grova, C. (2014) Optimal optode montage on electroencephalography/functional near-infrared spectroscopy caps dedicated to study epileptic discharges. J Biomed Opt 19(2), 026010. doi: 10.1117/1.JBO.19.2.026010.
